# Augmenting the accuracy of trainee doctors in diagnosing skin lesions suspected of skin neoplasms in a real-world setting: A prospective controlled before-and-after study

**DOI:** 10.1371/journal.pone.0260895

**Published:** 2022-01-21

**Authors:** Young Jae Kim, Jung-Im Na, Seung Seog Han, Chong Hyun Won, Mi Woo Lee, Jung-Won Shin, Chang-Hun Huh, Sung Eun Chang

**Affiliations:** 1 Department of Dermatology, Asan Medical Center, Ulsan University College of Medicine, Seoul, Korea; 2 Department of Dermatology, Seoul National University, Bundang Hospital, Seongnam, Korea; 3 I Dermatology, Clinic, Seoul, Korea; 4 IDerma, Inc, Seoul, Korea; Indian Institute of Technology Patna, INDIA

## Abstract

**Background:**

Although deep neural networks have shown promising results in the diagnosis of skin cancer, a prospective evaluation in a real-world setting could confirm these results. This study aimed to evaluate whether an algorithm (http://b2019.modelderm.com) improves the accuracy of nondermatologists in diagnosing skin neoplasms.

**Methods:**

A total of 285 cases (random series) with skin neoplasms suspected of malignancy by either physicians or patients were recruited in two tertiary care centers located in South Korea. An artificial intelligence (AI) group (144 cases, mean [SD] age, 57.0 [17.7] years; 62 [43.1%] men) was diagnosed via routine examination with photographic review and assistance by the algorithm, whereas the control group (141 cases, mean [SD] age, 61.0 [15.3] years; 52 [36.9%] men) was diagnosed only via routine examination with a photographic review. The accuracy of the nondermatologists before and after the interventions was compared.

**Results:**

Among the AI group, the accuracy of the first impression (Top-1 accuracy; 58.3%) after the assistance of AI was higher than that before the assistance (46.5%, *P =* .008). The number of differential diagnoses of the participants increased from 1.9 ± 0.5 to 2.2 ± 0.6 after the assistance (*P <* .001). In the control group, the difference in the Top-1 accuracy between before and after reviewing photographs was not significant (before, 46.1%; after, 51.8%; *P =* .19), and the number of differential diagnoses did not significantly increase (before, 2.0 ± 0.4; after, 2.1 ± 0.5; *P =* .57).

**Conclusions:**

In real-world settings, AI augmented the diagnostic accuracy of trainee doctors. The limitation of this study is that the algorithm was tested only for Asians recruited from a single region. Additional international randomized controlled trials involving various ethnicities are required.

## Introduction

For specific quantifiable problems, artificial intelligence (AI) has demonstrated performance comparable with that of specialists in the medical field [1]. In particular, convolutional neural networks (CNN) that mimic the structure of the retina have been widely used in medical image analysis.

In dermatology, AI could analyze dermoscopic and clinical images as accurately as dermatologists in reader tests [2–8]. However, these studies were all retrospective and mostly reader-tested for selected cases, which have complicated translation to actual practices for several limitations. First, the difference in diagnostic efficiency between algorithms and dermatologists was determined using experimental reader tests with limited clinical information related to the photographed skin abnormalities. The automated algorithms usually trained using data with limited relevancy, therefore, these algorithms may have practical limitations [9]. Second, AI model may not be trained using the characteristic feature of targeted disorders. One of the famous non-medical examples was “Clever Hans” phenomenon that the classifier discerns between huskies and wolves solely by the identification of a snowy background rather than real differences between huskies and wolves [10, 11]. Lastly, because algorithm fundamentally always predicted incorrect answers for the untrained cases, clinical evaluation for the uncertainty should be addressed in the prospective manner [12].

We have developed a skin disease classifier (Model Dermatology; https://modelderm.com) to diagnose 178 skin diseases and predict the chance of malignancy in previous studies [5, 13, 14]. At first, the algorithm was trained using 12 benign and malignant nodules for the classification of the most common skin neoplasms (build 2017) [13]. Because several benign disorders can mimic skin neoplasms, the algorithm should be a unified classifier that can predict 174 class disorders (build 2018) [5]. Further, because numerous trivial conditions may result in uncertainty of the algorithm, a large training dataset of the algorithm was created with the assistance of the region-based convolutional neural networks (build 2019; https://b2019.modelderm.com) [15].

A few algorithms have been tested in a prospective real-world setting where the expertise of the user affects the accuracy [16], and there is little data on whether the algorithm’s decision can really lead to a change in the clinician’s decision. In this study, we aimed to investigate whether the accuracy, sensitivity, and specificity of trainees improved with the assistance of an algorithm in real-world practice.

## Materials and methods

### Training of the algorithm

The training history of our algorithm (Model Dermatology; http://modelderm.com) was described in previous studies [5, 9, 12, 15, 17]. Image crops of normal and benign disorders were annotated based on the image findings and these image crops were used for the training to reduce false positives for common benign disorders. The classifier of the algorithm was trained with 721,749 image crops of 178 disease classes. With NVIDIA Caffe (https://github.com/nvidia/caffe; version 0.17.2, CUDA 10.0, cuDNN 7.6.2), we trained our CNN models using a transfer learning method using ImageNet pretrained models. Histogram normalization was performed as a preprocessing step before training the models. The output values of SE-Net [18] and SE-ResNeXt-50 were arithmetically averaged to obtain a final model output.

Along with three potential diagnoses, the algorithm reports a malignancy score (range: 0~100) using the following formula: Malignancy score = (basal cell carcinoma output + squamous cell carcinoma (SCC) output + SCC in situ output + keratoacanthoma output + malignant melanoma output) × 100 + (actinic keratosis output + ulcer output) × 20.

The algorithm reports an overall risk of malignancy as “Low”, “Medium,” or “High”. The algorithm reports the risk of malignancy as “Low” when the malignancy score is below 10, “Medium” when the score is between 10 and 20, and “High” when the score is over 20.

### Validation of the algorithm

After obtaining approval from the institutional review board of Asan Medical Center (2018–1130), a prospective study was performed at two tertiary care centers in Korea (230 cases from Department of Dermatology, Asan Medical Center, and 55 cases from Seoul National University, Bundang Hospital) between February 1, 2020, and November 7, 2020. The algorithm (Model Dermatology, build 2019; https://b2019.modelderm.com) developed in our previous study [5, 15] was used. The algorithm suggests the three most probable diagnosis of uploaded photographs and also reports a malignancy score (range: 0–100) (Supplementary Methods).

**Fig 1:**
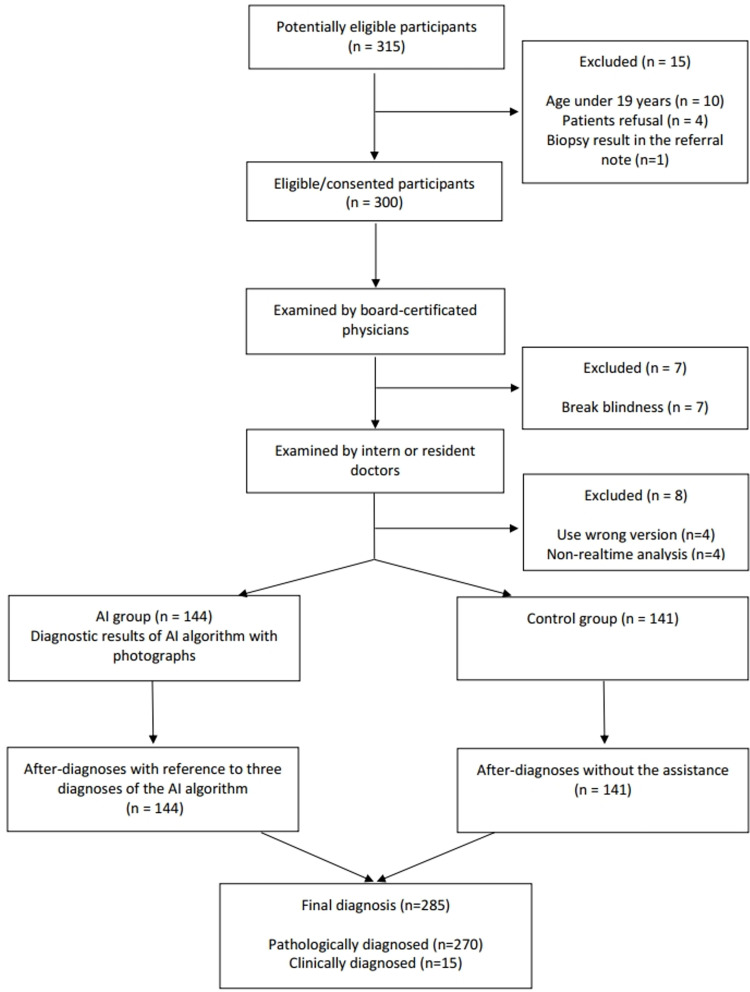
Study flowchart.

After obtaining informed consent, all patients (age > 19 years) who had skin neoplasms suspected of malignancy by either patient or physician were recruited. Exclusion criteria were patient refusal, broken blindness, the wrong version of the algorithm, non-real-time analysis, and exposure of the biopsy results in the referral note (Fig 1). If first impressions were recorded at >24 h after patients’ visits, they were classified as non-real-time. There were no inconclusive cases in the prediction of the algorithm. Ultimately, 270 pathologically diagnosed cases and 15 clinically diagnosed cases were used in the final analysis (Table 1 and S1 Table). A total of 139 and 131 cases were pathologically diagnosed in the AI group and the control group, respectively. A total of 15 cases (5 cases = AI group, 10 cases = Control group) were clinically diagnosed because the attending physicians concluded that they were definitely benign cases and do not to be biopsied.

**Table 1:** Dataset and demographic information.

	AI Group	Control Group
No. of Cases	144	141
Age (mean ± SD)	57.0 ± 17.7	61.0 ± 15.3
Males (%)	62 (43.1%)	52 (36.9%)
Onset*	6.9 ± 11.6	5.8 ± 9.3
Family history of skin cancer (+)	4 (2.8%)	5 (3.5%)
Tenderness (+)	16 (11.1%)	13 (9.2%)
Consistency (range 1–4)**	2.5 ± 0.9	2.6 ± 1.0
Suspicion		
by Patients (%)	79 (57.2%)	74 (54.0%)
by Physicians (%)	47 (32.6%)	48 (34.0%)
Location		
Head and neck	56 (38.9%)	65 (46.1%)
Trunk	42 (29.2%)	32 (22.7%)
Arm	15 (10.4%)	17 (12.1%)
Leg	30 (20.8%)	27 (19.1%)
Method of the diagnosis		
Pathologic diagnosis	139 (96.5%)	131 (92.9%)
Clinical diagnosis	5 (3.5%)	10 (7.1%)
Malignancy	23 (16.0%)	29 (20.6%)
Angiosarcoma	1	1
Basal cell carcinoma	7	18
Squamous cell carcinoma	6	5
Squamous cell carcinoma in situ	7	2
Keratoacanthoma	1	0
Melanoma	0	1
Metastasis	1	1
Mycosis fungoides	0	1
Benign (%)***	121 (84.0%)	112 (79.4%)

* Onset were available in 93.3% of cases (266 cases).

** The consistency was annotated as follows: 1 = hard, 2 = renitent, 3 = normal, and 4 = soft.

*** The details of the benign conditions are listed in the S1 Table.

A total of 10 attending physicians (11.4 ± 8.8 years’ experience after board certification), 11 dermatology trainees, and 7 intern doctors participated in this study (S2 Table). Attending physicians routinely recorded their diagnoses after thorough examinations. The trainees who were blinded to attending physicians’ diagnoses evaluated the patients. After quasirandomization using odd/even patient ID, the trainee took the patient’s medical history, performed physical examinations, took photographs, and provided their diagnoses up to three predictions. In the AI group, trainees selected one photograph and uploaded on http://b2019.modelderm.com. After referring to the algorithm’s three diagnoses and the malignancy score, they were given an opportunity to modify their initial diagnoses. In the control group, trainees just reviewed the photographs once again then provided the after-diagnoses.

Top accuracy was calculated as an evaluating metric. Top-(n) accuracy is the accuracy of the Top-(n) diagnoses. If any one of the Top-(n) diagnoses is correct, it counts as “correct.” Only an exact diagnosis was recorded as correct. For evaluating the sensitivity and specificity of malignancy prediction, the physicians’ diagnoses were transformed into either malignant or benign. Top accuracies were compared using two-tailed paired Wilcoxon signed-rank tests (R version 3.5.3), and a *P* value of < .05 was considered statistically significant.

## Results

### Result of the AI group

**Fig 2:**
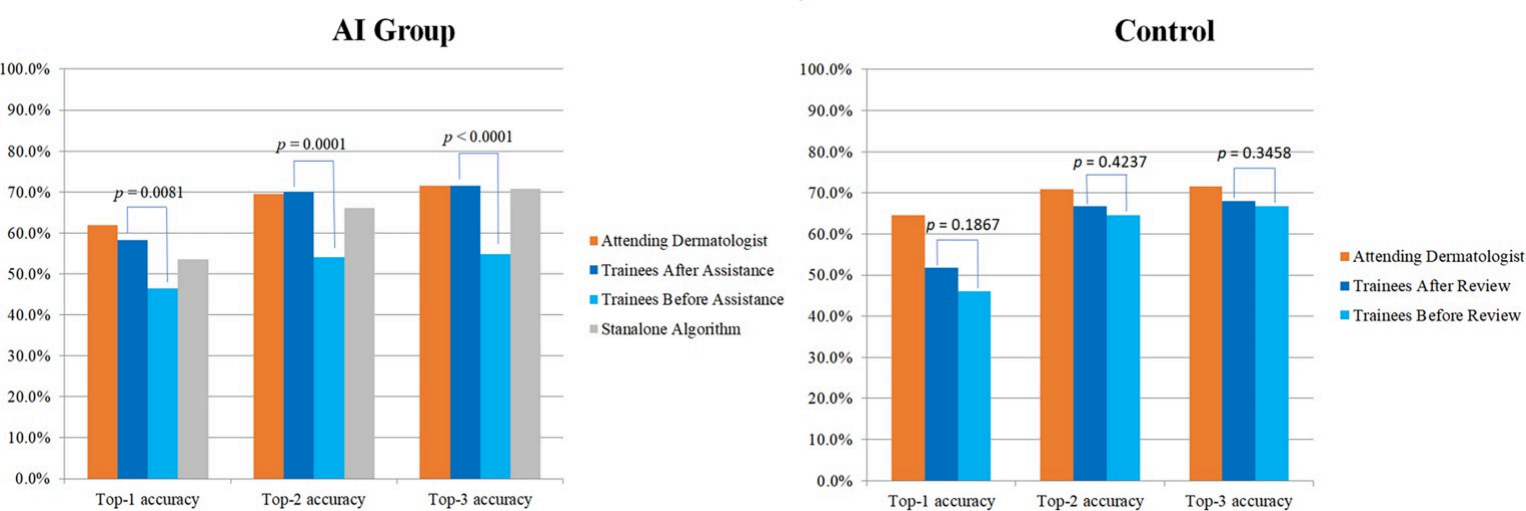
Top accuracies for diagnosing exact diseases. The physicians of the AI group (*n* = 144) referred to the three predictions of the algorithm’s diagnoses and the malignancy score before modifying their first impressions. The physicians of the Control group (*n* = 141) just reviewed the photographs once again. The *P*-values of top accuracies between before and after assistance of the trainees are annotated.

After analyzing the accuracies before and after assistance, it was noted that the Top-1/Top-2/Top-3 accuracies after assistance were significantly higher than those before assistance (before = 46.5%/ 54.2%/ 54.9%; after = 58.3%/ 70.1%/ 71.5%; *P =* .008/ < .001/ < .001) (Fig 2).

The Top-1/Top-2/Top-3 accuracies of the attending dermatologists were 61.8%/ 69.4%/ 71.5%, respectively, and those of the standalone algorithm were 53.5%/ 66.0%/ 70.8%, respectively. In 42.4% (61/144) cases, the Top-1 diagnosis of the algorithm was coherent with that of the trainees, and in 50.0% (72/144) cases, the Top-1 of the algorithm was coherent with that of the attending physicians. The Top-1 of the trainees was coherent with that of the attending physicians in 52.8% (76/144) cases.

The trainees revised 28.5% (41/144) of their Top-1 diagnosis after reviewing three diagnoses of the algorithm. A total of 70% (29/41) of their revised answers were correct, whereas 29% (12/41) of their revised answers were incorrect.

For determining malignancy, the sensitivity/specificity derived from the Top-1 was 78.3%/ 88.4% before the assistance and 73.9%/ 94.2% after the assistance (Table 2, *P =* .77/ = .06). The sensitivity/specificity of the attending dermatologists was 82.6%/ 91.7% and that of the patients were 56.5%/ 42.6%. The sensitivity/specificity derived from the Top-1 diagnosis of the algorithm was 52.2%/ 93.4%. The sensitivity/specificity at the threshold of the risk “Medium” using the malignancy score was 95.7%/ 60.3% and that at the threshold of the risk “High” was 82.6%/ 70.2% (Table 2).

**Table 2:** Summaries of the sensitivity and specificity.

		Sensitivity			Specificity		
		Before	after	P value	before	after	P value
AI Group	Top-1 of Trainees	78.3% (18/23)	73.9% (17/23)	0.7656	88.4% (107/121)	94.2% (114/121)	0.0572
	Top-2 of Trainees	87.0% (20/23)	91.3% (21/23)	0.7728	66.9% (81/121)	76.0% (92/121)	0.0289
	Top-3 of Trainees	95.7% (22/23)	91.3% (21/23)	0.7728	62.0% (75/121)	73.6% (89/121)	0.0085
	Top-1 of Attending Dermatologists	82.6% (19/23)		-	91.7% (111/121)		-
	Top-2 of Attending Dermatologists	95.7% (22/23)		-	82.6% (100/121)		-
	Top-3 of Attending Dermatologists	95.7% (22/23)		-	79.3% (96/121)		-
	Patients	56.5% (13/23)		-	42.6% (49/115)		-
	Top-1 of the algorithm	52.2% (12/23)		-	93.4% (113/121)		-
	Top-2 of the algorithm	69.6% (16/23)		-	78.5% (95/121)		-
	Top-3 of the algorithm	78.3% (18/23)		-	66.1% (80/121)		-
	Risk “High” of the algorithm	82.6% (19/23)		-	70.2% (85/121)		-
	Risk “Medium” of the algorithm	95.7% (22/23)		-	60.3% (73/121)		-
Control	Top-1 of Trainees	65.5% (19/29)	65.5% (19/29)	1.0000	81.3% (91/112)	86.6% (97/112)	0.0915
	Top-2 of Trainees	93.1% (27/29)	93.1% (27/29)	N/A	51.8% (58/112)	57.1% (64/112)	0.0411
	Top-3 of Trainees	93.1% (27/29)	93.1% (27/29)	N/A	49.1% (55/112)	53.6% (60/112)	0.1096
	Top-1 of Attending Dermatologists	79.3% (23/29)		-	90.2% (101/112)		-
	Top-2 of Attending Dermatologists	86.2% (25/29)		-	82.1% (92/112)		-
	Top-3 of Attending Dermatologists	86.2% (25/29)		-	79.5% (89/112)		-
	Patients	48.1% (13/27)		-	44.5% (49/110)		-

N/A: exact *p*-values with zeros could be computed.

The number of differential diagnoses by the trainees increased from 1.9 ± 0.5 to 2.2 ± 0.6 (*P <* .001).

### Result of the control group

The differences of the Top-1/Top-2/Top-3 accuracies between before and after reviewing photographs were not significant (Control-Before, 46.1%/ 64.5%/ 66.7%; Control-After, 51.8%/ 66.7/ 68.1%; *P =* .19/ = .42/ = .35).

For determining malignancy, the sensitivity/specificity derived from the Top-1 diagnosis was 65.5%/ 81.3% before reviewing and 65.5%/ 86.6% after reviewing (Table 2, *P =* 1.00/ = .09). The sensitivity/specificity of the attending dermatologists was 79.3%/ 90.2% and that of the patients was 48.1%/ 44.5%.

The number of differential diagnoses by the trainees had not changed significantly (Control-Before = 2.0 ± 0.4, Control-After = 2.1 ± 0.5; *P =* .57).

### AI group versus control group

The differences of the Top-1/Top-2/Top-3 accuracies between the AI group and the Control were not significant (AI Group = 58.3%/ 70.1%/ 71.5%; Control Group = 51.8%/ 66.7%/ 68.1%; *P =* .27/ = .53/ = .53). Summarized key results were described in S4 Table.

## Discussion

In this prospective study, we found that the AI assistance improved the diagnostic accuracy of trainee doctors. Owing to various biases, the outstanding performance of algorithms may not always be reproduced in real-world settings [16, 19]. Because algorithms cannot be trained for all diseases, they may show false positives for various out-of-distributed conditions. Both the metadata and photographs used in training and reader testing could be biased if handled by different expertise. For example, dermatologists may take few photographs of nail hematoma because they diagnose it with full confidence, and the algorithm trained with a few cases of hematoma may show uncertainty. Therefore, clinical validation should be performed with the same level of expertise as the end-user.

To date, the incorporations of AI into dermatological practice have been steadily investigated [2–8]. It was revealed that a trained classifier algorithm could execute diagnostic performance as equal as dermatologists for clinical and dermoscopic images of suspected melanoma and carcinoma [2]. Haenssle et al. [20] demonstrated that AI could correctly classify dermoscopic images of suspected melanoma into benign, in situ, or invasive at levels equal to and greater than expert dermatologists. Another recent study found that the performance of AI trained with dermoscopic images for identifying melanoma showed dermatologist-level image classification on a clinical image classification task. The mean sensitivity and specificity achieved by the 145 dermatologists with clinical images was 89.4% and 64.4%, whereas AI showed a mean specificity of 68.2% at the same sensitivity [3].

In our previous study, we also found that trained AI could classify clinical images into 12 common cutaneous diseases including skin neoplasms (basal cell carcinoma, squamous cell carcinoma, intraepithelial carcinoma, actinic keratosis, seborrheic keratosis, malignant melanoma, melanocytic nevus, lentigo, pyogenic granuloma, hemangioma, dermatofibroma, and wart) with similar sensitivity and specificity of dermatologists [5].

Reflecting these points on the diagnostic excellence of AI, the concept of augmented intelligence has recently emerged. Augmented intelligence is a term that focuses on the assistive role of AI, emphasizing that augmented intelligence is designed to enhance human intelligence and the clinician-patient relationship rather than substitute it [21]. The American medical association (AMA) states that augmented intelligence algorithms should be clinically validated before being integrated into patient care [22]. Therefore, they strongly recommended performing prospective clinical trials evaluating safety and effectiveness with relevant clinical end points. Despite these recommendations, previous studies incorporating AI into dermatological practice have not been prospectively verified in the real-world setting.

In this study, although the Top-1 accuracy of the standalone algorithm (53.5%) was comparable with that of the trainees (46.5%), the Top-1 accuracy of the augmented trainees (58.3%) was significantly higher. This augmentation could be owing to different strategies between humans and CNNs [23, 24]. The coherence between the algorithm–human (algorithm–trainees = 42.4%; algorithm–attending dermatologists = 50.0%) was lower than that between human–human (trainees–attending dermatologists = 52.8%), which implied different diagnostic patterns.

The augmentation may be achieved when the accuracy of the algorithm is higher or at least comparable with that of the user. In the study using dermoscopic images, the physicians with the least experience were the most frequently augmented [25]. For neoplastic skin lesions, the diagnostic accuracy of nondermatologists has been reported to be 40%–47% [26]. Experience improved the accuracy of plastic surgery trainees from 53.5% to 65.0% (21.5% increase) over a year of training [27]. In this study, the Top-1 accuracy of the trainees improved from 46.5% to 58.3% (25.4% increase) instantly by referring to the second opinion of the algorithm.

The sensitivity derived from the Top-1 prediction of the algorithm was low (52.2%), as noted previously [17]. Consequently, the sensitivity of the trainees derived from the Top-1 may decrease from 78.3% to 73.9% (*P =* .76). Our algorithm was developed with numerous benign crops to cope with the false-positive problem in detecting skin cancer using unprocessed images [15] and a multitude of benign crops in the training dataset could distort the overall output trend, making it more likely to predict benign conditions. The strong point of our study is that our algorithm also reported the malignancy score cut-off thresholds (“Low,” “Medium,” and “High” risk) to maintain appropriate sensitivity, unlike previous studies conducted without such complementary points.

### Limitation

Considering that our study population was limited to Asians, our results cannot be generalized in other circumstances. In completely different settings (Asian versus various races, tertiary care versus teledermatology, and Korea versus Chile as shown in our previous study [12]), the standalone accuracy of our algorithm was slightly lower than that of general physicians, although the algorithm could help increase the confidence of the dermatologists [12]. Because the prediction of the algorithm greatly relies on the characteristics of the training data, it may exhibit uncertainty in different settings. Deep learning-based algorithms reflect morphological features and even disease prevalence of the trained dataset; thus, algorithms show the best performance in the same environment. Indeed, the diagnostic performance of dermatologists may also be less accurate for patients belonging to non-local populations where a deep neural network trained with non-local populations may be expected to help close the gap [28].

We could not demonstrate the superiority of the AI Group over the Control Group in the manner of the randomized controlled trial. There was not a power and sample size calculation before initiating the study. Patients were randomly recruited but were not recruited consecutively. In addition, the two groups were not truly comparable.[29] As shown in S1 Table, the cases of BCC and SCC in situ were not assigned evenly, and as shown in S2 Table, the intern doctors with the least experience were more assigned to the AI Group.

## Conclusion

In the real-world setting, the standalone performance of the algorithm was comparable with that of the trainees, although the performance of the algorithm was reported to be comparable with dermatologists in the artificial setting [9]. Nevertheless, our algorithm could augment the accuracy of trainees in diagnosing suspected skin neoplasms by providing second opinions in real-time and increase the number of differential diagnoses in this prospective study. Further international randomized controlled trials are required to clarify the generalizability of the algorithm in other ethnicities and regions.

## Supporting information

S1 Table Dataset and demographic information. (DOCX)

S2 Table Number of examined cases and the grade of the participants. (DOCX)

S3 Table Top accuracies for the multiclass prediction. (DOCX)

S4 Table Summarized key results. (DOCX)

S5 Table Result of decision change. (DOCX)

S6 Table 178 Disorders trained on the algorithm in this study. (DOCX)

S1 File (XLSX)

S2 File (PDF)
