# Supplementary material for: Augmenting the accuracy of trainee doctors in diagnosing skin lesions suspected of skin neoplasms in a real-world setting: A prospective controlled before-and-after study
Source: PLoS One. 2022 Jan 21;17(1):e0260895. doi: 10.1371/journal.pone.0260895 (PMC8782525; doi:10.1371/journal.pone.0260895)
Supplement: S1 Table — (DOCX) [file pone.0260895.s001.docx]

**S1 Table. Dataset and demographic information.**

|  | **AI Group** | **Control Group** |
| --- | --- | --- |
| Malignancy | 23 (16.0%) | 29 (20.6%) |
| angiosarcoma | 1 | 1 |
| basal cell carcinoma | 7 | 18 |
| squamous cell carcinoma | 6 | 5 |
| squamous cell carcinoma in situ | 7 | 2 |
| keratoacanthoma | 1 | 0 |
| melanoma | 0 | 1 |
| metastasis | 1 | 1 |
| mycosis fungoides | 0 | 1 |
|  |  |  |
| Benign | 121 (84.0%) | 112 (79.4%) |
| abscess | 0 | 2 |
| actinic cheilitis | 1 | 1 |
| actinic keratosis | 7 | 8 |
| dermatofibroma | 10 | 6 |
| eczema | 2 | 3 |
| epidermal cyst | 4 | 5 |
| epidermal nevus | 1 | 0 |
| erythema nodosum | 0 | 2 |
| foreign body reaction | 1 | 0 |
| fungal infection | 0 | 2 |
| hemangioma | 6 | 5 |
| keloid/scar | 0 | 3 |
| lentigo | 3 | 1 |
| lipoma | 1 | 0 |
| melanocytic nevus | 29 | 27 |
| melanonychia | 1 | 0 |
| mucous cyst | 2 | 1 |
| neurofibroma | 1 | 2 |
| pigmented purpuric dermatosis | 1 | 0 |
| poroid hidradenoma | 0 | 1 |
| poroma | 2 | 1 |
| porokeratosis | 1 | 0 |
| postinflammatory hyperpigmentation | 1 | 2 |
| rosacea | 0 | 1 |
| schwannoma | 1 | 0 |
| sebaceous hyperplasia | 1 | 0 |
| sebaceoma | 0 | 1 |
| seborrheic keratosis | 36 | 31 |
| skin tag | 1 | 1 |
| subcorneal hemorrhage | 1 | 0 |
| wart | 4 | 4 |
| xanthogranuloma | 1 | 0 |
| unspecific pathologic diagnosis | 2 | 2 |
