# Supplementary material for: Augmenting the accuracy of trainee doctors in diagnosing skin lesions suspected of skin neoplasms in a real-world setting: A prospective controlled before-and-after study
Source: PLoS One. 2022 Jan 21;17(1):e0260895. doi: 10.1371/journal.pone.0260895 (PMC8782525; doi:10.1371/journal.pone.0260895)
Supplement: S2 Table — (DOCX) [file pone.0260895.s002.docx]

**S2 Table. Number of examined cases and the grade of the participants.**

|  | **AI Group** | **Control Group** |
| --- | --- | --- |
| Intern (n=7) | 69 | 46 |
| R1 (n=3) | 49 | 65 |
| R2 (n=4) | 14 | 18 |
| R3 (n=2) | 5 | 10 |
| R4 (n=2) | 7 | 2 |
| Total | 144 | 141 |

The R1 represents dermatology the first year resident trainee.
