# Supplementary material for: Augmenting the accuracy of trainee doctors in diagnosing skin lesions suspected of skin neoplasms in a real-world setting: A prospective controlled before-and-after study
Source: PLoS One. 2022 Jan 21;17(1):e0260895. doi: 10.1371/journal.pone.0260895 (PMC8782525; doi:10.1371/journal.pone.0260895)
Supplement: S3 Table — (DOCX) [file pone.0260895.s003.docx]

**S3 Table. Top accuracies for the multiclass prediction.**

| **AI-Group** | **Top-1 accuracy** | **Top-2 accuracy** | **Top-3 accuracy** |
| --- | --- | --- | --- |
| Attending Dermatologist | 61.8% (89/143) | 69.4% (100/143) | 71.5% (103/143) |
| Trainees After Assistance | 58.3% (84/143) | 70.1% (101/143) | 71.5% (103/143) |
| Trainees Before Assistance | 46.5% (67/143) | 54.2% (78/143) | 54.9% (79/143) |
| Standalone Algorithm | 53.5% (77/143) | 66.0% (95/143) | 70.8% (102/143) |
| **Control** | **Top-1 accuracy** | **Top-2 accuracy** | **Top-3 accuracy** |
| Attending Dermatologist | 64.5% (91/141) | 70.9% (100/141) | 71.6% (101/141) |
| Trainees After Review | 51.8% (73/141) | 66.7% (94/141) | 68.1% (96/141) |
| Trainees Before Review | 46.1% (65/141) | 64.5% (91/141) | 66.7% (94/141) |
