# Supplementary material for: Augmenting the accuracy of trainee doctors in diagnosing skin lesions suspected of skin neoplasms in a real-world setting: A prospective controlled before-and-after study
Source: PLoS One. 2022 Jan 21;17(1):e0260895. doi: 10.1371/journal.pone.0260895 (PMC8782525; doi:10.1371/journal.pone.0260895)
Supplement: S4 Table — (DOCX) [file pone.0260895.s004.docx]

| AI group | Before assistance | After assistance | *p*-value |
| --- | --- | --- | --- |
| Accuracy of the first impression  Top-1, % | 46.5 | 58.3 | *0.008* |
| Top-2, % | 54.2 | 70.1 | *<0.001* |
| Top-3, % | 54.9 | 71.5 | *<0.001* |
| Determination of malignancy  Sensitivity, % | 78.3 | 73.9 | *0.77* |
| Specificity, % | 88.4 | 94.2 | *0.06* |
| Number of differential diagnoses | 1.9 ± 0.5 | 2.2 ± 0.6 | *<0.001* |
|  |  |  |  |
| Control group |  |  |  |
| Accuracy of the first impression  Top-1, % | 46.1 | 51.8 | *0.19* |
| Top-2, % | 64.5 | 66.7 | *0.42* |
| Top-3, % | 66.7 | 68.1 | *0.35* |
| Determination of malignancy  Sensitivity, % | 65.5 | 65.5 | *1.00* |
| Specificity, % | 81.3 | 86.6 | *0.09* |
| Number of differential diagnoses | 2.0±0.4 | 2.1±0.5 | *0.57* |

**S4 Table. Summarized key results.**
