# Supplementary material for: Augmenting the accuracy of trainee doctors in diagnosing skin lesions suspected of skin neoplasms in a real-world setting: A prospective controlled before-and-after study
Source: PLoS One. 2022 Jan 21;17(1):e0260895. doi: 10.1371/journal.pone.0260895 (PMC8782525; doi:10.1371/journal.pone.0260895)
Supplement: S5 Table — (DOCX) [file pone.0260895.s005.docx]

**S5 Table. Result of decision change.**

| Cases that trainees changed their top-1diagnosis | | | | | | Total cases | | | |
| --- | --- | --- | --- | --- | --- | --- | --- | --- | --- |
| 41 (28.5%) | | | | | | 8 (5.6%) | 20 (13.8%) | 27 (18.7%) | 15  (10.4%) |
| Correct top-1 diagnosis after the aid of AI algorithm | | | Incorrect top-1 diagnosis after the aid of AI algorithm | | |  |  |  |  |
| 29 (20.1%) | | | 12 (8.3%) | | |  |  |  |  |
| 26 (18.1%) | 28 (19.4%) | 28 (19.4%) | 8 (5.6%) | 4 (2.8%) | 3 (2.1%) |  |  |  |  |
| Correctly changed top-1 diagnosis after the aid of correct top-1 diagnosis of AI | Correctly changed top-1 diagnosis after the aid of correct top-2 diagnosis of AI | Correctly changed top-1 diagnosis after the aid of correct top-3 diagnosis of AI | Incorrectly changed top-1 diagnosis after the aid of incorrect top-1 diagnosis of AI | Incorrectly changed top-1 diagnosis after the aid of incorrect top-2 diagnosis of AI | Incorrectly changed top-1 diagnosis after the aid of incorrect top-3 diagnosis of AI | Incorrect top-1 diagnosis despite correct top-1 diagnosis of AI | Incorrect top-1 diagnosis despite correct top-2 diagnosis of AI | Incorrect top-1 diagnosis despite correct top-3 diagnosis of AI | Correct top-1 diagnosis despite incorrect top-1 diagnosis of AI |
