# Supplementary material for: Augmenting the accuracy of trainee doctors in diagnosing skin lesions suspected of skin neoplasms in a real-world setting: A prospective controlled before-and-after study
Source: PLoS One. 2022 Jan 21;17(1):e0260895. doi: 10.1371/journal.pone.0260895 (PMC8782525; doi:10.1371/journal.pone.0260895)
Supplement: S6 Table — (DOCX) [file pone.0260895.s006.docx]

**S6 Table. 178 Disorders trained on the algorithm in this study.**

| 1 | ABNOM |
| --- | --- |
| 2 | Abscess |
| 3 | Acanthosis nigricans |
| 4 | Acne |
| 5 | Acne scar |
| 6 | Actinic keratosis |
| 7 | Acute generalized exanthematous pustulosis |
| 8 | Alopecia areata |
| 9 | Amyloidosis |
| 10 | Androgenic alopecia |
| 11 | Anetoderma |
| 12 | Angioedema |
| 13 | Angiofibroma |
| 14 | Angiokeratoma |
| 15 | Angular cheilitis |
| 16 | Atopic dermatitis |
| 17 | Basal cell carcinoma |
| 18 | Becker nevus |
| 19 | Blue nevus |
| 20 | Bullous pemphigoid |
| 21 | Burn |
| 22 | Cafe au lait macule |
| 23 | Callus |
| 24 | Cellulitis |
| 25 | Cheilitis |
| 26 | Cherry Hemangioma |
| 27 | Chronic eczema |
| 28 | Condyloma |
| 29 | Confluent reticulated papillomatosis |
| 30 | Congenital nevus |
| 31 | Contact dermatitis |
| 32 | Cutaneous horn |
| 33 | Cyst |
| 34 | Depressed scar |
| 35 | Dermal melanosis |
| 36 | Dermatofibroma |
| 37 | Dilated pore |
| 38 | Drug eruption |
| 39 | Dysplastic nevus |
| 40 | Eccrine hidrocystoma |
| 41 | Eczema herpeticum |
| 42 | Epidermal cyst |
| 43 | Epidermal nevus |
| 44 | Erythema ab igne |
| 45 | Erythema annulare centrifugum |
| 46 | Erythema multiforme |
| 47 | Erythema nodosum |
| 48 | Exfoliative dermatitis |
| 49 | Fifth disease |
| 50 | Folliculitis |
| 51 | Fordyce spot |
| 52 | Freckle |
| 53 | Furuncle |
| 54 | Granuloma annulare |
| 55 | Guttate psoriasis |
| 56 | Halo nevus |
| 57 | Hand eczema |
| 58 | Hemangioma |
| 59 | Hematoma |
| 60 | Herpes simplex |
| 61 | Herpes zoster |
| 62 | Herpetic whitlow |
| 63 | Hypertrophic scar |
| 64 | Idiopathic guttate hypomelanosis |
| 65 | Impetigo |
| 66 | Infantile eczema |
| 67 | Inflammed cyst |
| 68 | Ingrowing nail |
| 69 | Insect bite |
| 70 | Intraepithelial carcinoma (Bowen disease) |
| 71 | Irritate fibroma |
| 72 | Irritated lentigo or seborrheic keratosis |
| 73 | Juvenile xanthogranuloma |
| 74 | Keloid |
| 75 | Keratoacanthoma |
| 76 | Keratoderma |
| 77 | Keratosis pilaris |
| 78 | Lentigo |
| 79 | Lichen nitidus |
| 80 | Lichen planus |
| 81 | Lichen simplex chronicus |
| 82 | Lichen striatus |
| 83 | Livedo reticularis |
| 84 | Livedoid vasculitis |
| 85 | Lupus erythematosus |
| 86 | Lymphangioma |
| 87 | Malignant melanoma |
| 88 | Melanocytic nevus |
| 89 | Melanonychia |
| 90 | Melasma |
| 91 | Milia |
| 92 | Molluscum contagiosum |
| 93 | Morphea |
| 94 | Mucocele |
| 95 | Mucosal melanotic macule |
| 96 | Mucous cyst |
| 97 | Nail dystrophy |
| 98 | Neurofibroma |
| 99 | Neurofibromatosis |
| 100 | Nevus depigmentosus |
| 101 | Nevus spilus |
| 102 | Nipple eczema |
| 103 | Nonspecific (normal) |
| 104 | Normal nail |
| 105 | Nummular eczema |
| 106 | Onycholysis |
| 107 | Onychomysosis |
| 108 | Organoid nevus |
| 109 | Ota nevus |
| 110 | Palmoplantar pustulosis |
| 111 | Panniculitis |
| 112 | Papular urticaria |
| 113 | Parapsoriasis |
| 114 | Paronychia |
| 115 | Perioral dermatitis |
| 116 | Periungual fibroma |
| 117 | Photosensitive dermatitis |
| 118 | Pigmented progressive purpuric dermatosis |
| 119 | Pitted keratolysis |
| 120 | Pityriasis alba |
| 121 | Pityriasis lichenoides chronica |
| 122 | Pityriasis lichenoides et varioliformis acuta |
| 123 | Pityriasis rosea |
| 124 | Poikiloderma |
| 125 | Pompholyx |
| 126 | Porokeratosis |
| 127 | Poroma |
| 128 | Portwine stain |
| 129 | Postinflammatory hyperpigmentation |
| 130 | Prurigo nodularis |
| 131 | Prurigo pigmentosa |
| 132 | Psoriasis |
| 133 | Purpura |
| 134 | Pustular psoriasis |
| 135 | Pyoderma gangrenosum |
| 136 | Pyogenic granuloma |
| 137 | Riehl melanosis |
| 138 | Rosacea |
| 139 | Scabies |
| 140 | Scar |
| 141 | Sebaceus hyperplasia |
| 142 | Seborrheic dermatitis |
| 143 | Seborrheic keratosis |
| 144 | Senile gluteal dermatosis |
| 145 | Senile purpura |
| 146 | Skin tag |
| 147 | Soft fibroma |
| 148 | Squamous cell carcinoma |
| 149 | Staphylococcal scalded skin syndrome |
| 150 | Steatocystoma multiplex |
| 151 | Striae distensae |
| 152 | Subungual hematoma |
| 153 | Syphilis |
| 154 | Syringoma |
| 155 | Systemic contact dermatitis |
| 156 | Tattoo |
| 157 | Telangiectasia |
| 158 | Tinea corporis |
| 159 | Tinea cruris |
| 160 | Tinea faciei |
| 161 | Tinea pedis |
| 162 | Tinea versicolor |
| 163 | Toxic epidermal necrosis |
| 164 | Ulcer |
| 165 | Urticaria |
| 166 | Urticaria pigmentosa |
| 167 | Urticarial vasculitis |
| 168 | Varicella |
| 169 | Vascular malformation |
| 170 | Vasculitis |
| 171 | Venous lake |
| 172 | Verruca plana |
| 173 | Viral exanthem |
| 174 | Vitiligo |
| 175 | Wart |
| 176 | Xanthelasma |
| 177 | Xanthoma |
| 178 | Xerotic eczema |
