# Supplementary material for: Augmenting the accuracy of trainee doctors in diagnosing skin lesions suspected of skin neoplasms in a real-world setting: A prospective controlled before-and-after study
Source: PLoS One. 2022 Jan 21;17(1):e0260895. doi: 10.1371/journal.pone.0260895 (PMC8782525; doi:10.1371/journal.pone.0260895)
Supplement: S2 File — (PDF) [file pone.0260895.s008.pdf]

주소 : 05505 서울특별시 송파구 올림픽로 43길 88 서울아산병원 TEL : 02-3010-7166, FAX : 02-3010-4163

심의결과 통지서

심의결과 통지일	2020년 10월 23일	심의방법	◉ 정규 ○ 신속

접수번호	S2018-1703-0010					
과제번호	2018-1130					
과제명	Deep Learning 기술을 이용한 피부암 진단의 전향적 연구					
연구책임자	소속	피부과	직위	교수	성명	장성은
의뢰자	소속	IIT				
연구상세분류	생명윤리법	인간대상연구				
	연구대상	검사법				
	연구구분	설문조사연구				
	연구단계					
심의종류	연구계획변경					
심의결과	연구개시 및 지속, 변경사항 적용이 가능한 결과	☑ 승인	☐ 기존대로 연구지속			
	보완심의 또는 이의 신청이 필요한 결과	☐ 시정승인	☐ 보완(조건부)			
		☐ 보완(재심의)	☐ 기각			
		☐ 연구는 지속하나 보완 필요	☐ 연구는 지속하나 새로운 연구대상자 모집 중지			
		☐ 연구는 지속하나 이후 연구대상자에게 이루어지는 연구절차 중지	☐ 승인된 연구의 일시중지			
		☐ 승인된 연구의 조기종료	☐ 연구자에 대한 조치			
		☐ 반려	☐ 기타			
		☐ 보완				

서류접수일	2020년 10월 12일	심의일	2020년 10월 15일
지속심의주기	☐ 3개월 ☐ 6개월 ☑ 1년<br>☐ 면제 ☐ 기타	승인유효기간	2021년 07월 19일

주소 : 05505 서울특별시 송파구 올림픽로 43길 88 서울아산병원 TEL : 02-3010-7166, FAX : 02-3010-4163

기타 심의 의견
본 위원회에서는 연구자가 제출하신 연구계획변경을 심의한 결과 승인하기로 결정하였습니다.<br><br>변경내용:<br>1. 연구계획서<br>2. 연구대상자 설명문 및 동의서<br>3. 연구책임자 (문익준 → 장성은)<br>4. 공동연구자 (장성은 → 문익준)

제출자료 목록 및 버전번호
연구계획서(국문)(1.4)
연구대상자 설명문 및 동의서(1.3)

임상연구심의위원회/기관생명윤리위원회	위원장 이무송

본 임상연구심의위원회는 국제표준화추진회의(ICH), 의약품임상시험관리기준/의료기기임상시험실시기준(KGCP) 및 생명윤리및안전에관한법률 등 관련 법규를 준수합니다. 본 연구와 이해상충관계가 있는 위원이 있을 경우 해당 위원은 연구의 심의에서 배제하였습니다.
